# Association between CD14 Gene C-260T Polymorphism and Inflammatory Bowel Disease: A Meta-Analysis

**DOI:** 10.1371/journal.pone.0045144

**Published:** 2012-09-26

**Authors:** Zhengting Wang, Jiajia Hu, Rong Fan, Jie Zhou, Jie Zhong

**Affiliations:** 1 Department of Gastroenterology, Ruijin Hospital, Shanghai Jiaotong University School of Medicine, Shanghai, China; 2 Department of Nuclear Medicine, Ruijin Hospital, Shanghai Jiaotong University School of Medicine, Shanghai, China; University of Aberdeen, United Kingdom

## Abstract

**Background:**

The gene encoding CD14 has been proposed as an IBD-susceptibility gene with its polymorphism C-260T being widely evaluated, yet with conflicting results. The aim of this study was to investigate the association between this polymorphism and IBD by conducting a meta-analysis.

**Methodology/Principal Findings:**

Seventeen articles met the inclusion criteria, which included a total of 18 case-control studies, including 1900 ulcerative colitis (UC) cases, 2535 Crohn's disease (CD) cases, and 4004 controls. Data were analyzed using STATA software. Overall, association between C-260T polymorphism and increased UC risk was significant in allelic comparison (odds ratio [OR]  = 1.21, 95% confidence interval [CI]: 1.02–1.43; P = 0.027), homozygote model (OR  = 1.44, 95% CI: 1.03–2.01; P = 0.033), as well as dominant model (OR  = 1.36, 95% CI: 1.06–1.75; P = 0.016). However, there was negative association between this polymorphism and CD risk across all genetic models. Subgroup analyses by ethnicity suggested the risk-conferring profiles of -260T allele and -260 TT genotype with UC in Asians, but not in Caucasians. There was a low probability of publication bias.

**Conclusions/Significance:**

Expanding previous results of individual studies, our findings demonstrated that *CD14* gene C-260T polymorphism might be a promising candidate marker in susceptibility to UC, especially in Asians.

## Introduction

Crohn's disease (CD) and ulcerative colitis (UC) are the two major subtypes of inflammatory bowel disease (IBD), which is a chronic relapsing and remitting inflammatory condition affecting the gastrointestinal tract. Familial aggregation and twin studies reported that patients with IBD carried strong genetic predisposition [1]. Recent genome-wide association studies (GWAS) have identified approximately 100 IBD-susceptibility loci, and thereof more than 70 loci susceptible to CD and 47 to UC have been confirmed in subsequent meta-analyses, especially in the genes encoding microbe recognition, lymphocyte activation, cytokine signaling, and intestinal epithelial defense [2]–[6]. The results of GWAS provided new insights into the immunopathogenesis of this disease, implicating an important role of the innate and adaptive immune systems in disease occurrence.

Toll-like receptors (TLRs) are a class of proteins that are active in innate and adaptive systems. TLRs are members of a conserved interleukin (IL)-1 superfamily of transmembrane receptors that recognize pathogen-associated molecular patterns (PAMPs) which present on the surface of pathogens. The pathogenesis of IBD, ulcerative colitis and Crohn's disease may be due to increased TLR or decreased TLR signalling respectively [7]. TLR4−/− mice increased susceptibility to bleeding and bacterial translocation in DSS (dextran sodium sulfate)-induced colitis model [8], [9]. CD14 serves as a receptor for lipopolysaccharides (LPS), and the binding of LPS/CD14 complex to TLR4 could activate NF-κB, and further induce an inflammatory response [10]. Soluble CD14 accompanying with serum LPS-binding protein are markers of disease activity in patients with Crohn's disease [11]. Co-existence of a mutation in either TLR4 or CD14 gene, and in NOD2/CARD15 is associated with an increased susceptibility to developing CD compared to UC, and to developing either CD or UC compared to healthy individuals [12], [13]. It is required for the microbe-induced endocytosis of TLR4. In dendritic cells, this CD14-dependent endocytosis pathway is upregulated upon exposure to inflammatory mediators [14]. Meanwhile, human *CD14* gene is mapped on chromosome 5q31.1, adjacent to a region reportedly in linkage with IBD [15], [16]. From genetic perspective, the substitution of a promoter polymorphism in *CD14* gene (C-260T, also known as *CD14*. C-159T) results in elevated transcriptional activity and accordingly high serum CD14 levels [17], [18]. Therefore, candidacy of *CD14* for IBD is well-defined and its C-260T polymorphism has been reported to be associated with IBD by some but not all studies [19], [20]. Generally, studies with insufficient sample sizes account for such inconsistency [21].

**Figure 1:**
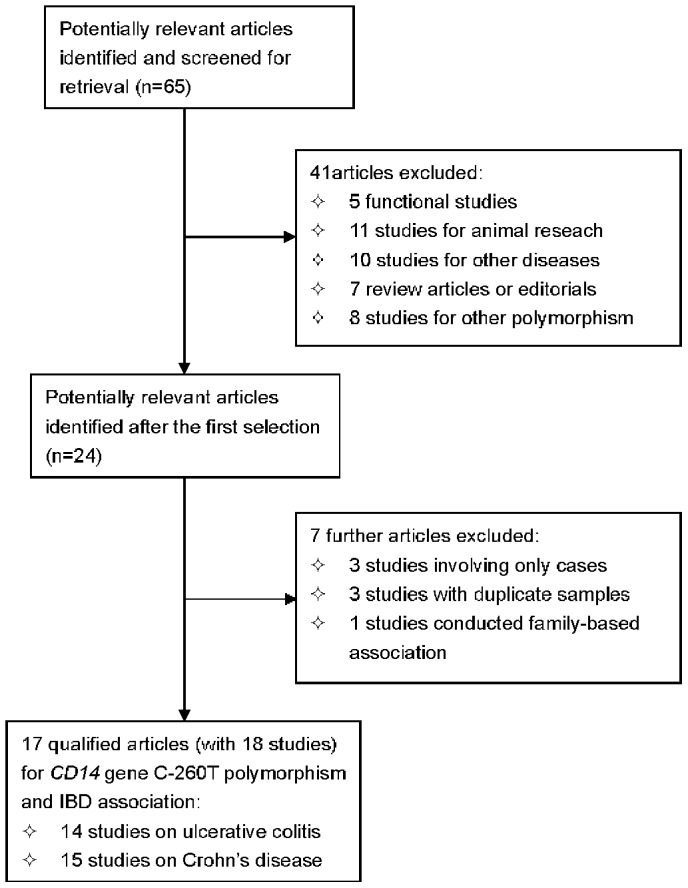
Flow diagram of search strategy and study selection.

To shed some light on this issue, we sought to examine the association of *CD14* gene C-260T polymorphism with the occurrence of IBD by a meta-analysis, and simultaneously to identify factors attributed to between-study heterogeneity and publication bias.

**Table 1:** The baseline characteristics of all eligible studies.

First author	Year	Ethnicity	Study design	Genotyping method	Numbers			T freq. (%)			HWE
					Cases		Controls	Cases		Controls	
					CD	UC		CD	UC		
Klein W et al.	2002	Germany (C)	NA	PCR-RFLP	219	142	410	51.1	43.7	44.5	0.9006
Obana N et al.	2002	Japan (A)	NA	PCR-RFLP	82	101	123	48.2	57.4	44.7	0.5234
Klein W et al.	2003	Germany (C)	HB	PCR-RFLP	253	/	650	51.0	/	44.5	0.993
Torok HP et al.	2004	Germany (C)	PB	PCR-RFLP	102	98	145	48.5	43.4	50.0	0.9983
Arnott IDR et al.	2004	UK (C)	PB	PCR-RFLP	242	233	189	49.0	52.8	51.9	0.6105
Klausz G et al.	2005	Hungary (C)	NA	Probe	133	/	75	48.5	/	46.0	0.9599
Guo QS et al.	2005	Chinese (A)	PB	PCR-RFLP	/	114	160	/	64.0	60.3	0.9989
Gazouli M et al.	2005	Greece (C)	HB	PCR-RFLP	120	85	100	50.4	40.0	37.0	0.6079
Leung E et al.	2005	New Zealand (C)	PB	PCR-RFLP	185	/	187	51.4	/	50.3	0.7974
Peters KE et al.	2005	Australia (C)	PB	PCR-RFLP	235	81	189	49.6	56.8	49.2	0.5584
Ouburg S et al.	2005	Netherlands (C)	HB	PCR-RFLP	112	/	170	44.6	/	47.7	0.9544
XUE H et al.	2007	Chinese (A)	HB	PCR-RFLP	41	43	135	61.0	60.5	59.3	0.6887
Wang F et al.	2007	Japanese (A)	HB	PCR-RFLP	/	97	135	/	66.0	48.5	0.5882
Baumgart DC et al.	2007	Hungary (C)	NA	Probe	144	118	202	42.7	44.1	52.7	0.7091
Baumgart DC et al.	2007	Germany (C)	NA	Probe	235	145	403	45.3	53.8	45.5	0.9415
Petermann I et al.	2009	New Zealand (C)	PB	Taqman	387	405	377	49.6	48.4	49.3	0.984
Sivaram G et al.	2012	India (A)	NA	PCR-RFLP	/	139	176	/	53.2	41.8	0.3855
Kim EJ et al.	2012	Korea (A)	HB	PCR-RFLP	45	99	178	61.1	54.6	36.5	0.8755

*Abbreviations*: HB  =  hospital-based design; PB  =  population-based design; NA  =  not available; CD  =  Crohn's disase; UC  =  ulcerative colitis.

## Methods

This meta-analysis is reported in accordance with the Preferred Reporting Items for Systematic Reviews and Meta-analyses (PRISMA) guideline (please see supplementary PRISMA checklist) [22].

**Figure 2:**
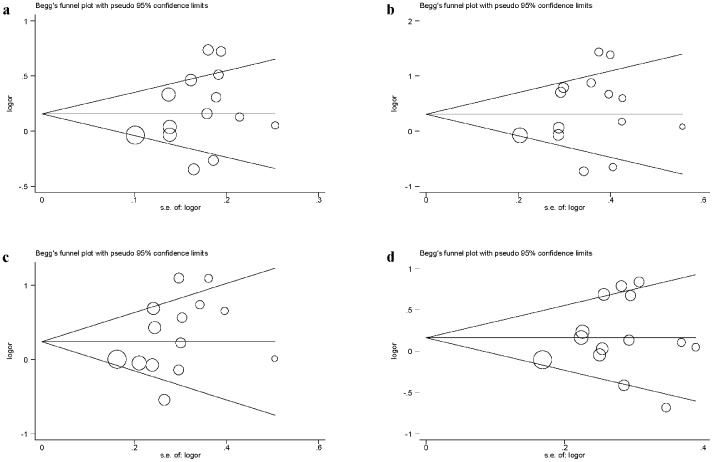
Begg's funnel plots of publication bias test for CD14 C-260T polymorphism with UC (a. -260T vs.-260C allele; b. -260TT vs. -260CC; c. dominant model; d. recessive model). Vertical axis represents the log of OR; horizontal axis represents the SE of log(OR). Funnel plots are drawn with 95% confidence limits. OR, odds ratio; SE, standard error. The graphic symbols represents the data in the plot be sized proportional to the inverse variance.

### Search strategy for identification of studies

We searched the PubMed, EMBASE, ISI Web of Knowledge databases and Wanfang databases before July 20, 2012, by using the key subjects “inflammatory bowel disease”, “IBD”, “Crohn's disease”, “CD”, “Ulcerative colitis”, “UC”, in combination with “Cluster of differentiation 14”, “CD14 ”, “toll like receptor 4”, “TLR4” and “TLR-4”. We read the full text of the retrieved articles to inspect whether data on the topic of interest were included. Reference lists of the retrieved articles and systematic reviews were also checked for citations of articles that were not initially identified. Special meeting issues of journals (Abstract only) were removed from the searching results. Search results were restricted to human populations and articles written in English or Chinese. If more than one geographic or ethnic heterogeneous group was reported in one report, each was extracted separately. If two or more studies shared the whole or part of study populations, the one with larger sample size was extracted.

**Table 2:** Subgroup analysis of CD14 C-260T gene polymorphisms and IBD (CD and UC).

Variables	Allele contrast		Homozygote model		Dominant model		Recessive model	
	OR (95% CI)	*p*	OR (95% CI)	*p*	OR (95% CI)	*p*	OR (95% CI)	*p*
**CD14 C-260T gene polymorphism and CD**								
**Total**	1.10(0.96,1.25)	0.167	1.18(0.92,1.52)	0.201	1.05(0.88,1.23)	0.603	1.20(0.97,1.49)	0.090
**Descent of populations**								
Caucasians	1.05(0.93,1.18)	0.479	1.08(0.85,1.38)	0.510	1.00(0.85,1.18)	0.991	1.12(0.91,1.38)	0.265
Asians	1.49(0.84,2.66)	0.171	2.03(0.70,5.91)	0.193	1.53(0.83,2.82)	0.169	1.77(0.76,4.13)	0.185
**Source of controls**								
HB	1.40(1.01,1.94)	0.046	1.82(1.00,3.32)	0.050	1.36(0.92,2.01)	0.127	1.67(1.05,2.65)	0.030
PB	0.99(0.88,1.11)	0.808	0.97(0.77,1.23)	0.801	0.96(0.79,1.16)	0.669	1.00(0.83,1.22)	0.970
NA	1.02(0.81,1.29)	0.879	1.03(0.64,1.65)	0.914	0.95(0.69,1.31)	0.761	1.10(0.72,1.69)	0.663
**Genotyping methods**								
PCR-based	1.18(1.00,1.38)	0.045	1.35(1.00,1.81)	0.049	1.12(0.93,1.36)	0.225	1.33(1.03,1.71)	0.027
Probe or Taqman	0.93(0.77,1.13)	0.459	0.86(0.58,1.26)	0.430	0.87(0.62,1.23)	0.444	0.93(0.69,1.24)	0.607
**CD14 C-260T gene polymorphism and UC**								
**Total**	1.21(1.02,1.43)	0.027	1.44(1.03,2.01)	0.033	1.36(1.06,1.75)	0.016	1.19(0.96,1.48)	0.112
**Descent of populations**								
Caucasians	1.01(0.87,1.19)	0.866	1.01(0.73,1.41)	0.938	1.09(0.83,1.43)	0.538	0.95(0.79,1.15)	0.626
Asians	1.58(1.28,1.95)	0.000	2.51(1.77,3.55)	0.000	1.97(1.46,2.65)	0.000	1.69(1.27,2.25)	0.000
**Source of controls**								
HB	1.54(1.08,2.20)	0.017	2.27(1.11,4.66)	0.025	1.92(1.12,3.31)	0.018	1.65(1.10,2.48)	0.016
PB	1.03(0.88,1.20)	0.754	1.09(0.73,1.61)	0.679	1.16(0.85,1.58)	0.354	0.95(0.75,1.20)	0.674
NA	1.20(0.88,1.63)	0.251	1.37(0.77,2.45)	0.287	1.24(0.80,1.91)	0.336	1.25(0.85,1.85)	0.258
**Genotyping methods**								
PCR-based	1.29(1.06,1.56)	0.009	1.63(1.12,2.36)	0.010	1.48(1.13,1.94)	0.005	1.30(1.01,1.68)	0.044
Probe or Taqman	0.99(0.70,1.39)	0.956	0.98(0.48,2.00)	0.954	1.06(0.57,1.95)	0.863	0.93(0.68,1.28)	0.668

*Abbreviations*: OR  =  odds ratio; CI  =  confidence interval; HB  =  Hospital based; PB  =  Population based; NA  =  Not available.

### Inclusion/exclusion criteria

Identified studies satisfied the following criteria: (1) evaluation of *CD14* gene C-260T polymorphism with the risk for IBD (CD and/or UC); (2) case-control or cross-sectional or nested case-control study in design; (3) availability of genotype or allele counts of studied polymorphisms between patients and controls in order to estimate odds ratio (OR) and its corresponding 95% confidence interval (CI).

**Figure 3:**
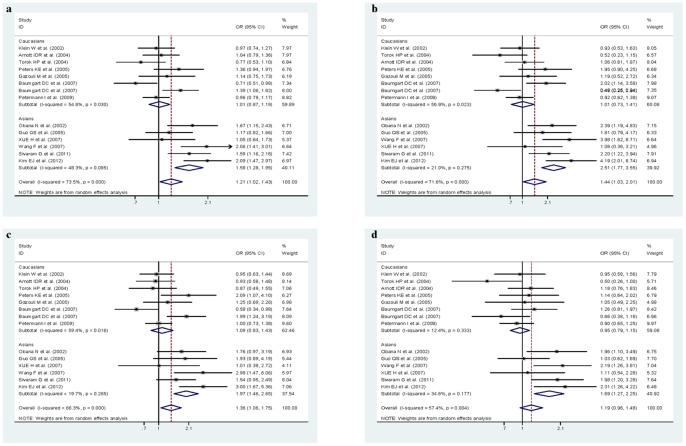
Subgroup analyses of CD14 C-260T polymorphism to UC by ethnicity ( a. -260T vs. -260C allele; b. -260TT vs. -260CC; c. dominant model; d. recessive model). There was significant association among Asian populations, whereas no substantive changes was observed in Caucasians in any kind of comparisons.

### Extracted information

The following data were extracted independently and entered into separate databases by two authors (Z. Wang and J. Hu) from each qualified study: first author's last name, publication year, population ethnicity, study design, genotyping methods, baseline characteristics of the study population including age, gender, subtype of the disease, and the genotype or allele distributions in patients and controls. Any encountered discrepancies were adjudicated by a discussion and an 100% consensus was reached.

### Statistical analysis

In this meta-analysis, we assessed the association of *CD14* gene C-260T polymorphism with IBD risk under allelic, homozygous and dominant and recessive models, respectively. Crude OR and 95% CI were calculated to compare contrasts of alleles or genotypes between patients and controls.

Deviation from Hardy-Weinberg equilibrium was assessed using Pearson χ^2^ test. The random-effects model using DerSimonian & Laird method was employed to bring the individual effect-size estimates together irrespective of between-study heterogeneity [23]. Heterogeneity was evaluated by the *I* ^2^ statistic, which was documented for the percentage of the observed between-study variability due to heterogeneity rather than chance with its values ranging 0–100% [*I* ^2^ = 0–25%, no heterogeneity; *I* ^2^ = 25–50%, moderate heterogeneity; *I* ^2^ = 50–75%, large heterogeneity; *I* ^2^ = 75–100%, extreme heterogeneity [24]. In the case of between-study heterogeneity, we examined the study characteristics that can stratify the studies into subgroups with homogeneous effects. Subgroup analyses were conducted after stratifying studies performed on various ethnic/geographic populations or studies with different study designs (hospital-based and population-based) or studies on different subtype diseases. Here, the design of studies was determined according to the sources of control group, either from hospitals or general populations.

Finally, evidence for publication bias was assessed using Egger's test and visual funnel plot inspection. The Egger's test detects funnel plot asymmetry by determining whether the intercept deviates significantly from zero in a regression of the standardized effect estimates against their precision.

Probability less than 0.05 was judged significant with the exception of the *I* ^2^ statistic and publication tests, where a significance level of less than 0.1 was chosen. Statistical analyses were performed using STATA version 11.0 for Windows (StataCorp LP, College Station, Texas, USA).

## Results

### Search results

The detailed selection process is presented in Figure 1. Based on our search strategy, the primary screening yielded 65 potentially relevant articles. Because the study by Baumgart DC et al included two populations, we treated them separately in this meta-analysis. Therefore, seventeen articles met the inclusion criteria, which included a total of 18 case-control studies. 17 articles met the inclusion criteria, which included a total of 18 case-control studies in an attempt to examine the association of *CD14* gene C-260T polymorphism with IBD [12], [13], [19], [20], [25]–[37]. A total of 4435 IBD cases (1900 UC and 2535 CD) and 4004 controls were finally analyzed. Of these 18 studies, seventeen [12], [13], [19], [20], [25]–[36] were published in English and one in Chinese [37]. Six studies were conducted in Asians (two in Chinese [20], [37], two in Japanese [28], [34], and one in India [31] and Korea [13], respectively), whereas 12 [12], [13], [19], [20], [25]–[36] studies were conducted in Caucasians.

### Study characteristics

The baseline characteristics of all qualified studies are presented in Table 1. The frequency of -260T allele was 51.71% in UC patients, 49.21% in CD patients and 47.29% in controls, with the higher frequency in average Asians cases than Caucasians cases (65.98% vs 48.47% in UC, 54.76% vs 48.82% in CD), whereas with similar frequency in controls (48.02% in Asians vs 47.08%). Genotyping for C-260T polymorphism across all studies, except three using the probe technology [25], [26] and one using TaqMan assay [36], was conducted using polymerase chain reaction-restriction fragment length polymorphism (PCR-RFLP) followed by enzyme ScaI digestion.

### Associations between CD14 polymorphism and UC

The pooled OR from all included studies indicated a significant association between *CD14* polymorphism and increased UC risk in allelic comparison (OR  = 1.21, 95% CI: 1.02–1.43; P = 0.027) with low possibility of publication bias as reflected by the suggestive asymmetry of funnel plot (Figure 2) and the Egger's test (P = 0.25), although there was strong evidence of between-study heterogeneity (*I^2^* = 73.5%, P<0.001). The magnitude of OR in allele comparison was similar to the homozygote (OR  = 1.44, 95% CI: 1.03–2.01; P = 0.033) as well as dominant models (OR  = 1.36, 95% CI: 1.06–1.75; P = 0.016), while no significant association was found in recessive model (OR  = 1.19, 95% CI: 0.96–1.48, P = 0.112).

Considering the fact that study design, the ethnicity difference, as well as the method of genotype might attribute to the sources of heterogeneity, we conducted separate analyses according to these factors.

In view of study design, no obvious association existed in the population-based subgroup and NA group, but significant association between *CD14* C-260T polymorphism and UC risk was observed across all genetic models, in the hospital-based subgroup (Table 2).

Further subgroup analysis by ethnicity suggested heterogeneous associations of C-260T polymorphism with UC, by showing that there was significant association among Asian populations, whereas no substantive changes was observed in Caucasians in any kind of comparisons (Figure 3).

To evaluate the possible effect of genotyping methods on the variability of overall estimates, studies were divided into PCR-based subgroup and Taqman or Probe subgroup, and importantly the magnitude of association in PCR-based studies was reinforced with the -260T allele conferring a significant risk effect on UC (OR  = 1.29; 95% CI: 1.06–1.56; P = 0.009), whereas this effect was reversed in Taqman or Probe subgroup studies with no attainable significance (OR  = 0.99; 95% CI: 0.70–1.39; P = 0.956).

### Associations between CD14 polymorphism and CD

Overall no significant association was found for *CD14* C-260T polymorphism in allele comparison (OR  = 1.10, 95% CI: 0.96–1.25; P = 0.167), homozygote (OR = 1.18, 95% CI: 0.92–1.52, P = 0.201), dominant model (OR  = 1.05, 95% CI: 0.88–1.23, P = 0.603) and recessive model (OR  = 1.20; 95% CI: 0.97–1.49; P = 0.09), even in Asians or Caucasians stratified by ethnicity. However, subgroup analyses by study design detected significance for allele comparison (OR  = 1.40; 95% CI: 1.01–1.94; P = 0.046), homozygote model (OR  = 1.82; 95% CI: 1.00–3.32; P = 0.05) with increased CD risk, in hospital-based studies, even for the recessive models (OR  = 1.67; 95% CI: 1.05–2.65; P = 0.03). When we conducted an stratified analysis by the genotyping methods, there were obvious differences. For example, a increased risk was observed in allele comparison (OR  = 1.18, 95% CI: 1.00–1.38; P = 0.045) and homozygote model (OR  = 1.35, 95% CI: 1.00–1.81, P = 0.049) for PCR-based subgroup; whereas no materially change in odds was observed in Taqman or Probe subgroup studies (Table 2).

### Publication Bias

As reflected by the funnel plots (Figure 2) and the corresponding Egger's test, there was a low probability of publication bias for the *CD14* gene C-260T polymorphisms under study.

## Discussion

To the best of our knowledge, the present study involving 8439 subjects represents the first meta-analysis investigating the relationship between *CD14* gene C-260T polymorphism and risk for IBD. Our results demonstrated that -260T allele carriers were at moderate increased risk of developing UC, especially in Asians, although this finding might suffer from the disturbance of significant heterogeneity. Moreover, differences in ethnicity and study design were identified as potential sources of heterogeneity. Furthermore, the relatively large samples examined and low probability of publication bias as reflected by visual inspection of the funnel plots along with Egger's tests indicated the robustness of our results.

It is inevitable to encounter genetic heterogeneity in any disease identification strategy [38]. As exemplified in the present study, frequency of -260T allele in patients differed remarkably between Caucasians and Asians (65.98% vs. 48.47% in UC, 54.76% vs. 48.82% in CD), leaving open the question that divergent genetic backgrounds or linkage disequilibrium patterns may account for this difference. Meanwhile, the possibility of *CD14* gene C-260T being in close linkage with different nearby causal variants in different populations cannot be excluded. Additionally, it is widely believed that genetic markers in predisposition to IBD vary across geographical and racial groups. As evidenced, nucleotide oligomerization domain (NOD)2/caspase-activation recruitmentdomains (CARD)-15 polymorphisms have been strongly associated with CD in Caucasians [39], [40], but not in individuals of Asian descent [41], [42]. Accordingly, in our ethnicity-stratified analyses, *CD14* gene C-260T polymorphism exhibited remarkable heterogeneity with UC across ethnic groups, with significance attained in Asians but not in Caucasians, suggesting that *CD14* C-260T might exert a pleiotropic impact in the pathogenesis of UC or interact with other genetic or environmental factors. Furthermore, CD14 is a monocytic differentiation antigen that regulates innate immune responses to pathogens. *CD14* C-260T polymorphism has been reported to be associated with some immune-related diseases, such as allergic rhinitis [43], pediatric asthma [44] and juvenile idiopathic arthritis [45], most in Asians [46]. These results are similar to ours, and may provide a hint for exploring the complex relationship between CD14 genetic polymorphisms, ethnic difference and susceptibility to human immune-related diseases. To unravel this uncertainty, more and more large, well-designed studies are required to understand the genetic variability of IBD.

Besides the disturbing influence of ethnicity in this meta-analysis, it should still be treated with caution that differences in study design constituted another potential source of heterogeneity. Although positive association of *CD14* gene C-260T polymorphism with UC risk was observed across all genetic models in hospital-based studies, we run the risk of overestimating this association in view of the striking weaknesses of this type of design, such as population stratification and admixture. To obtain convincing evidence, well-designed studies with less error-prone methods are encouraged.

So far, as two major subtypes of IBD, CD and UC are believed to share overlapping but distinct clinical and pathological features, and have great differences in etiology and genetic backgrounds [47], [48]. In this study, we found that carriers of -260T allele were at moderate increased risk of developing UC, but not in CD, indicating the different roles of *CD14* polymorphism in the IBD different subgroups. Given the insufficient study power in each subgroup, much more research within the framework of genetics and biology is warranted.

Despite the clear strengths of our study including relatively large sample sizes and robustness of statistical analyses, interpretation of our current study, however, should be viewed in light of several technical limitations. First, all included studies were cross-sectional in design, which precludes us to make inference on causality. Second, because only published studies were retrieved and articles in languages other than English or Chinese were not included, publication bias might be possible, even though our funnel plots and statistical tests indicated no observable bias. Third, our results were based on unadjusted estimates. It seems likely that the *CD14* gene C-260T polymorphism individually make a moderate contribution to risk prediction in UC patients, but whether the polymorphism integrated with other risk factors will enhance the prediction requires additional research. Thus, a more precise analysis should be conducted with individual data, which would allow for the adjustment by other co-varieties such as age, gender, lifestyle and other genetic factors.

Taken together, we expand previously individual studies on IBD by suggesting that *CD14* gene C-260T polymorphism might contribute to the occurrence of UC, especially in Asians. Also our observations leave open the question regarding the heterogeneous effect of -260T allele across different ethnic populations. Nonetheless, for practical reasons, we hope that this study will not remain just another endpoint of research instead of a beginning to establish the background data for further investigation on pathphysiological mechanisms of *CD14* gene on IBD.

## Supporting Information

Text S1 **Checklist of items to include when reporting a systematic review or meta-analysis (diagnostic review consisting of cohort studies).**
(DOC)
